# WhatsApp and atopic dermatitis: a clinical trial

**DOI:** 10.1016/j.jped.2024.07.003

**Published:** 2024-08-08

**Authors:** Thaís Braga Cerqueira, Renata Robl Imoto, Mariana Muzzolon, Vânia Oliveira de Carvalho

**Affiliations:** a Programa de Pós-Graduação em Dermatologia Pediátrica, Universidade Federal do Paraná (UFPR), Curitiba, PR, Brazil; b Programa de Pós-Graduação em Saúde da Criança e do Adolescente, Complexo do Hospital de Clínicas, Universidade Federal do Paraná (UFPR), Curitiba, PR, Brazil

**Keywords:** Atopic dermatitis, Health education, Quality of life, Pediatrics, Text message

## Abstract

**Objective:**

To evaluate the effect of text messages with information about atopic dermatitis (AD) on the quality of life (QoL) of children and their caregivers and on the severity of the disease.

**Methods:**

Researcher-blinded randomized controlled clinical trial. The experimental group (EG) received messages about AD and the control group (CG) about general health. A total of 56 children under 15 years of age and their caregivers, allocated to the CG and EG, were assessed on admission, after one month, and after four months. Improvement in QoL was measured by the Children's Dermatology Life Quality Index (CDLQI), the Infants’ Dermatitis Quality of Life Index (IDQOL), and the Dermatitis Family Impact Questionnaire (DFIQ), and improvement in the severity of AD by the Scoring of Atopic Dermatitis (SCORAD) and the Eczema Area and Severity Index (EASI).

**Results:**

Median age was of nine years, 33 (58.9 %) were girls. The CG and EG had similar results, except for the higher frequency of mild AD in the CG and moderate/severe AD in the EG—these severity categories were kept grouped together. Regarding mild and moderate/severe AD in the EG, the SCORAD score decreased (*p* = 0.03 and *p* < 0.001). The EASI in both groups showed a significant reduction (mild AD: CG: *p* = 0.01, EG: *p* = 0.04; moderate/severe AD: CG: *p* = 0.05, EG: *p* = 0.02). The QoL of children and caregivers improved only in the EG (*p* = 0.01). Intergroup analysis showed no differences.

**Conclusion:**

The improvement in the severity of AD in both groups suggests the positive effects of educational interventions in general, not only those specific to the disease.

## Introduction

Atopic dermatitis (AD) is a chronic, relapsing dermatosis whose control depends on the implementation of an exhaustive treatment routine.^1^

The clinical presentation varies according to age group, with acute eczema on the malar area and trunk being more common in infants, while chronic eczema located in limb flexures, face, and neck is more frequent in schoolchildren and adolescents. In adults, AD phenotypes can vary and coexist in the same patient, such as the nodular prurigo pattern, hand dermatitis, and facial and neck dermatitis, which significantly impact the quality of life.^2^

Treatment involves the use of emollients, topical corticosteroids, calcineurin inhibitors, and even systemic treatments such as phototherapy and immunosuppressants for moderate to severe cases. The choice of therapeutic approach may vary depending on the clinical phenotype presented by the patient.^2^

Health education actions are an auxiliary measure for the success of AD treatment since the guidance provided during medical consultations may be insufficient to understand the disease and consequent adherence to treatment.^1^

Educating both caregivers and patients affects the quality of life (QoL) of children and adolescents with AD, reducing its severity, preventing complications, reducing costs, and coping better with the disease.^3^^,^^4^

Several modalities of therapeutic patient education (TPE) have proved effective in reducing severity or improving QoL, such as meetings,^5^, ^6^, ^7^ written materials,^8^ and videos.^9^^,^^10^ Singer et al. evaluated the effect of sending educational text messages about AD care on reducing the severity of the disease.^11^ However, few studies^12^ have addressed this issue, which, along with the lack of an ideal educational intervention model, hinders data comparison.

Given the possibility of increasing patient education about the disease and the scarcity of data,^12^ this study aimed to evaluate the use of text messages with information about AD, their influence on the QoL of children and their caregivers, and their effect on the severity of the disease.

## Materials and methods

This researcher-blinded randomized controlled clinical trial was conducted in a tertiary hospital in Brazil, from March 2022 to June 2023. It was approved by the institution's Human Research Ethics Committee (CAAE 36652220.9.0000.0096) and registered in the Brazilian Registry of Clinical Trials (ReBEC; RBR-3m97hhq). All participants signed an informed consent form.

### Sample

Children under 15 years of age who met the criteria for a clinical diagnosis of AD, with no other chronic diseases, except for mild asthma and rhinitis, whose caregivers had the WhatsApp® app on their cell phones. Participants who started using immunosuppressants up to two months before inclusion in the study were excluded. All participants were allocated to the control group (CG) or the experimental group (EG) by simple randomization using the Randomizer platform.

### Variables assessed for severity of AD and quality of life

All participants were assessed on admission, one month, and four months after the messages were sent. Clinical outcomes included children's QoL and the severity of AD.

The Children's Dermatology Life Quality Index *(*CDLQI; for children over five years of age), the Infants’ Dermatitis Quality of Life Index (IDQOL; for children up to five years of age), and the Dermatitis Family Impact Questionnaire (DFIQ; for guardians) evaluated the effect on QoL with domains related to symptoms and feelings, personal relationships, changes in routine activities, changes in sleep, and difficulties with treatment for the patient and family, respectively.

The IDQOL includes 10 questions covering: pruritus, the child's mood, sleep, leisure activities, problems during meals, problems caused by treatment, the level of comfort when dressing or undressing the child, and bath time problems. A separate additional question addresses the severity of AD, which is scored from none to extremely severe.^13^ The CDLQI includes 10 questions referring to different aspects of the child's life affected by the disease in the last week. It consists of six domains: symptoms and feelings, leisure, school or vacation, personal relationships, sleep, and treatment.^14^ The DFIQ has 10 questions on household chores, food preparation, sleep, family leisure, shopping, spending, tiredness, emotional stress, relationships, and the effect of helping with treatment on guardians’ lives.^15^ All questionnaires (IDQOL, CDLQI and DFIQ) refer to symptoms in the week before they were assessed. The score for each question ranges from 0 to 3 points, for a total of 30 points. The higher the score, the worse the QoL.

Scoring of Atopic Dermatitis (SCORAD) and the Eczema Area and Severity Index (EASI) assessed the extent, characteristics, and severity of dermatological lesions on physical examination. The SCORAD also measures pruritus and changes in sleep in the last 48 h before the assessment. The SCORAD score ranges from 0 to 103 and classifies AD as mild (0 to 25 points), moderate (26 to 50 points), and severe (greater than 50 points). The maximum EASI score is 72 and the severity of the disease is classified as none (0), very mild (0.1 – 1.0), mild (1.1 – 7.0), moderate (7.1 – 20), severe (20.1 – 50), and very severe (50.1 – 72).

To avoid inducing responses, participants read and answered the questionnaires by themselves.

### Therapeutic patient education intervention

The intervention consisted of sending text messages once a day to the caregiver's cell phone for four weeks. In the EG, the messages were related to AD: pathophysiology, triggering factors, and treatment of the disease; written in lay language, and sent four times a week for educational purposes and three times to encourage compliance with medical prescriptions. Participants in the CG received messages with information on general health, such as healthy eating, and the importance of physical exercise, among others (Table 1 - Supplementary Material).

The text messages were sent via WhatsApp® from a cell phone with an Android® operating system. The researcher who applied the severity and QoL assessment tools was blind to the messages.

Data were analyzed using Statistica 4.0 statistical software (StatSoft Power Solutions, Inc., Palo Alto, California, USA). The Mann-Whitney and Kruskal-Wallis ANOVA tests were used for asymmetric continuous variables, while Fisher's exact test, the Pearson chi-square test, and the McNemar test were used for categorical variables. Multivariate and multiple logistic regression models were used to identify the predictor variables for QoL and severity of AD.

The sample was estimated considering an effect size of variation of at least 2 points in the QoL score, a significance level of 5 %, and a type II error of 10 %, with an estimated sample of 28 cases in each group.

## Results

The study sample consisted of 60 participants. The authors excluded four because they did not receive regular messages due to changes in their WhatsApp® number without prior notice, or because they did not have daily internet access. The final sample included 56 participants, with a median age of nine years (IIQ 5–11.5), 33 of whom were girls (58.9 %). The CG and EG were similar in terms of age, sex, severity of AD, and QoL before the intervention (Table 1).

**Table 1:** Clinical characteristics of the control and experimental groups at the first assessment.

Characteristics	CG (*n* = 28)	EG (*n* = 28)	p
Female sex	17 (60.7 %)	16 (57.1 %)	1.00^a^
Age (years old)	7.0 (4.5–11)	9.0 (6–12)	0.45^b^
Exclusive topical treatment	25 (89 %)	24 (86 %)	1.00^a^
CDLQI/IDQOL score	5.0 (2–9)	7.0 (3.5–10.5)	0.32^b^
CDLQI/IDQOL classification			
No effect	4 (14.3 %)	2 (7.1 %)	
Weak effect	13 (46.4 %)	11 (39.3 %)	0.65^c^
Moderate effect	6 (21.4 %)	9 (32.1 %)	
Strong effect	5 (17.9 %)	5 (17.9 %)	
Very strong effect	0 (0.0 %)	1 (3.6 %)	
DFIQ score	5.5 (3.5–12.5)	10.0 (4.5–14.0)	0.27^b^
DFIQ classification			
No effect	4 (14.3 %)	3 (10.7 %)	
Weak effect	12 (42.9 %)	8 (28.6 %)	
Moderate effect	5 (17.9 %)	6 (21.4 %)	0.74^c^
Strong effect	5 (17.9 %)	8 (28.6 %)	
Very strong effect	2 (7.1 %)	3 (10.7 %)	
Severity of AD			
SCORAD	23.1 (14.9–31.5)	25.5 (18.8–38.3)	0.14
Mild	17 (60.7 %)	11 (39.3 %)	
Moderate	10 (35.7 %)	16 (57.1 %)	*0.17^a^
Severe	1 (3.6 %)	1 (3.6 %)	
EASI	1.6 (0.8–5.2)	1.7 (0.9–4.8)	0.69^b^
Very mild	9 (32.1 %)	8 (28.6 %)	
Mild	16 (57.1 %)	16 (57.1 %)	0.90^c^
Moderate	3 (10.7 %)	4 (14.3 %)	

CG, control group; EG, experiment group; CDLQI/IDQOL, Children's Dermatology Life Quality Index/Infants’ Dermatitis Quality of Life Index; DFIQ, Dermatitis Family Impact Questionnaire; AD, atopic dermatitis; SCORAD, Scoring of Atopic Dermatitis; EASI, Eczema Area and Severity Index.

a Fisher's exact test.

b Mann–Whitney test.

c Pearson chi-square test.

⁎ Considering mild and moderate categories.

When grouping together the moderate and severe categories, the authors found a higher frequency of mild AD in the CG (*n* = 17) and moderate/severe AD in the EG (*n* = 17). Therefore, the authors kept the moderate and severe categories grouped together in the subsequent assessment.

In both the CG and the EG, the AD treatment instituted on admission and in the follow-up showed no differences, with two participants in each group changing their treatment from only topical to associated with systemic at four months (*p* = 1.00). The systemic treatments used were methotrexate, cyclosporine, phototherapy, dupilumab, and upadacitinib.

**Figure 1:**
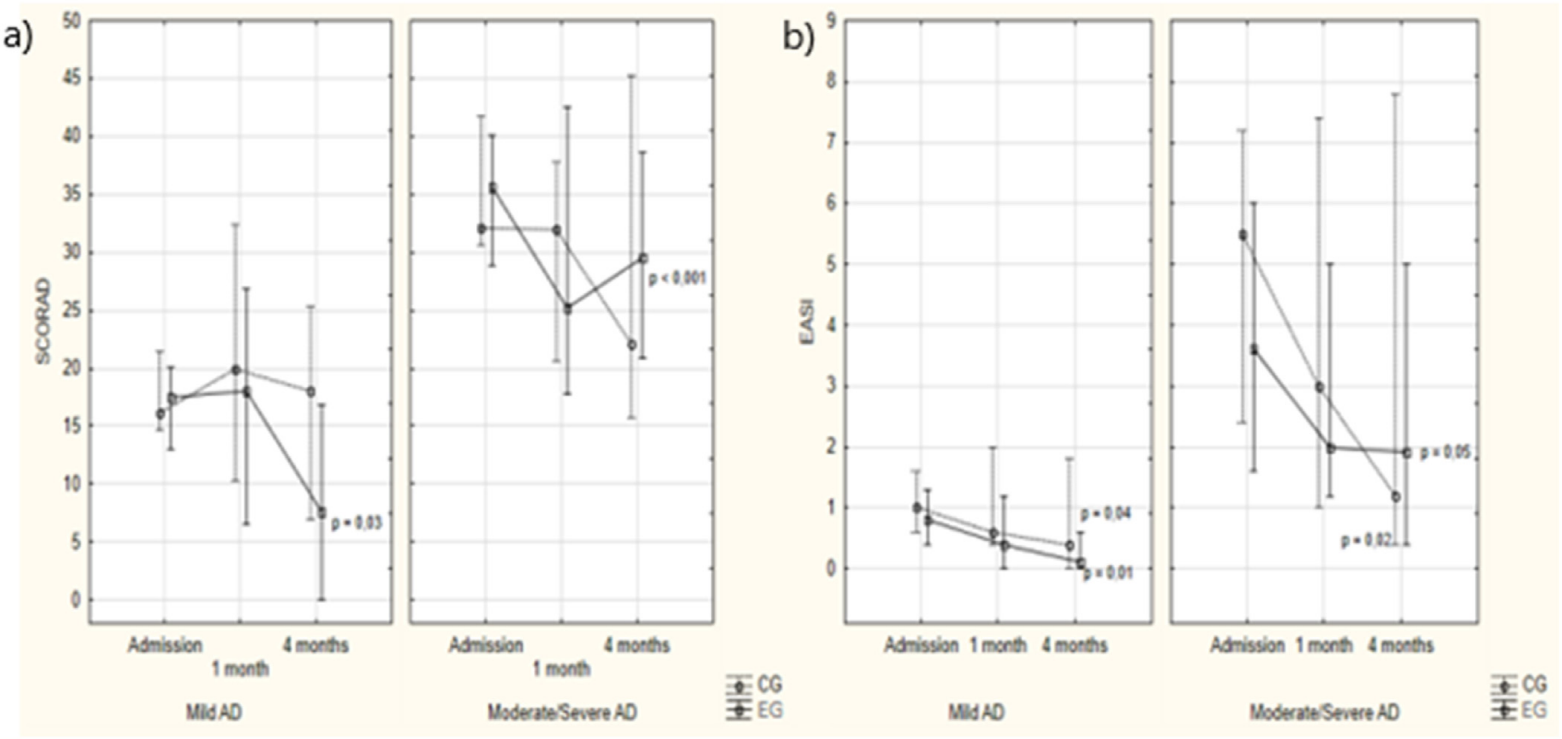
SCORAD and EASI according to study groups and assessment times. Note: Intergroup analysis: Kruskal-Wallis ANOVA – Mild AD: admission: *p* = 0.86; 1 month: *p* = 0.60; 4 months: *p* = 0.08. Moderate/severe AD: admission: *p* = 1.00; 1 month: *p* = 0.37; 4 months: *p* = 0.79. Intragroup analysis: Friedman ANOVA – Mild AD: CG: *p* = 0.83; EG: *p* = 0.03. Moderate/severe AD: CG: *p* = 0.52; **EG: *p*<0.001**. Nemenyi post-hoc test. NOTE: Intergroup analysis: Kruskal-Wallis ANOVA – Mild AD: admission: *p* = 0.58, 1 month: *p* = 0.18; 4 months: *p* = 0.16. Moderate/severe AD: admission: *p* = 0.60; 1 month: *p* = 0.79; 4 months: *p* = 0.98. Intragroup analysis: Friedman ANOVA – Mild AD: CG: *p* = 0.01; EG: *p* = 0.04. Moderate/severe AD: CG: *p* = 0.05; **EG: *p*=0.02**.

Regarding mild and moderate/severe AD in the EG, the SCORAD score decreased (*p* = 0.03 and *p* < 0.001). Among the 17 cases of mild AD on admission in the CG, 10 (58.8 %) remained unchanged and seven (41.2 %) worsened to moderate/severe AD by the fourth month. In the EG, all participants with mild AD remained with the same severity in the final assessment (*p* < 0.001; Figure 1).

Regarding moderate/severe AD, in the CG, of the 11 cases in this category upon admission, six (54.5 %) improved to mild AD, while five (45.5 %) worsened in disease severity. In the EG, among the 17 cases classified with moderate/severe disease, seven (41.2 %) improved to mild disease, and 10 (58.8 %) maintained the same disease severity. Thus, while there was a 45.5 % worsening in the CG, there was no worsening in the EG (41.2 % improved, *p* < 0.001).

Regarding the EASI score, both groups showed a significant reduction, with no difference between the groups at any time during the assessment. All were, in median, in the very mild or mild AD range (*p* = 0.58, *p* = 0.18, and *p* = 0.16, respectively; Figure 1).

**Figure 2:**
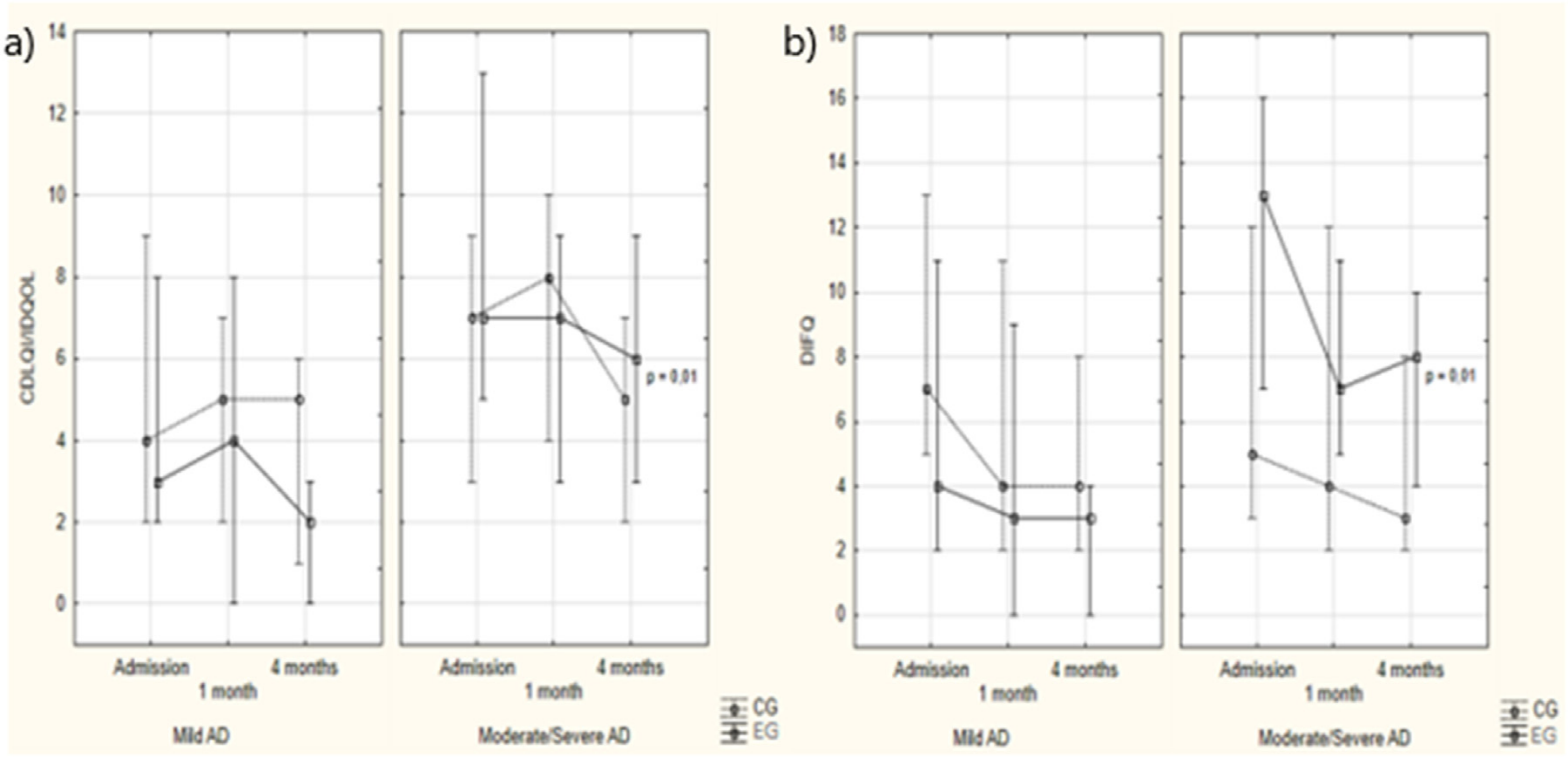
CDLQI/IDQOL and DFIQ values according to study groups and assessment times. (a) Note: Intergroup analysis: Kruskal-Wallis ANOVA – Mild AD: admission: *p* = 0.79; 1 month: *p* = 0.49; 4 months: *p* = 0.10. Moderate/severe AD: admission: *p* = 0.34; 1 month: *p* = 0.54; 4 months: *p* = 0.58. Intragroup analysis: Friedman ANOVA – Mild AD: CG: *p* = 0.67; EG: *p* = 0.19. Moderate/severe AD: CG: *p* = 0.17; **EG: *p*=0.01**. (b) Note: Intergroup analysis: Kruskal-Wallis ANOVA – Mild AD: admission: *p* = 0.23; 1 month: *p* = 0.32; 4 months: *p* = 0.13. Moderate/severe AD: admission: *p* = 0.12; 1 month: *p* = 0.63; 4 months: *p* = 0.24. Intragroup analysis: Friedman ANOVA – Mild AD: CG: *p* = 0.08; EG: *p* = 0.28. Moderate/severe AD: CG: *p* = 0.35; **EG: *p*=0.01**.

Regarding children's QoL, the CG showed no significant variation in the CDLQI/IDQOL score for mild AD (*p* = 0.67) or moderate/severe AD (*p* = 0.17). In the EG, the intervention did not change QoL in cases of mild AD (*p* = 0.19) but reduced it in moderate/severe cases (*p* = 0.01). In the CG, QoL improved in four cases (23.5 %), while 11 cases (64.7 %) showed no change and two cases worsened (11.8 %). In the EG, four cases showed improvement (36.4 %), five remained unchanged (45.4 %), and two (18.2 %) worsened, with no significant difference between the groups (*p* > 0.05). Regarding moderate/severe AD in the CG, QoL improved in four cases (36.4 %), while five cases showed no change (45.4 %) and one (9.1 %) worsened. In the EG, these frequencies were nine (52.9 %), seven (41.2 %), and one (5.9 %), with no statistically significant difference between the groups (*p* > 0.05; Figure 2).

Regarding guardians’ QoL, the DIFQ score only decreased significantly in the EG and in cases of moderate/severe AD (*p* = 0.01, Figure 2).

In the multivariate logistic regression model, considering the severity of AD as the outcome variable and intervention and systemic treatment as independent variables, systemic treatment had the greatest effect on the severity of the disease (OR 13.15; 95 % CI 1.43–18.73; *p* < 0.001). Considering children's QoL as the outcome variable and the severity of AD, intervention, and systemic treatment as independent variables, the severity of the disease was the main predictor of improved QoL (OR 3.62; 95 % CI 0.98–32.6; *p* = 0.04; Table 2).

**Table 2:** Multivariate logistic regression: outcome variables severity of atopic dermatitis and quality of life.

Outcome variable severity of AD			
Independent variables	OR	95 % CI	p
Intervention	1.56	0.24–10.13	0.61
Treatment	13.15	1.43–18.73	<0.001
Outcome variable QoL			
Independent variables	OR	95 % CI	p
Intervention	1.29	0.37–4.49	0.68
Treatment	2.72	0.59–12.51	0.19
Severity of AD	3.62	0.98–32.6	0.04

AD, atopic dermatitis; QoL, quality of life; OR, odds ratio; CI, confidence interval.

## Discussion

In this study, the severity of AD decreased according to the scores used and QoL improved throughout the assessments, with no differences between the CG and EG.

Few studies address the effects of educational interventions on the severity of AD and the QoL of children and their families. Moreover, the authors use different methodologies, including one-off individual^7^ and group^5^^,^^6^ sessions, informative written materials,^8^ videos,^9^^,^^10^^,^^16^ and biweekly groups from six weeks^17^ to six months.^18^^,^^19^ Singer et al. sent text messages about general skin care and AD triggers.^11^ Studies report positive effects of educational interventions on the severity of the disease^5^^,^^6^^,^^9^^,^^17^^,^^19^^,^^20^^,^^21^ and the QoL of children and caregivers.^18^^,^^22^

In the present study, when evaluating participants in the EG with mild AD, all remained in the same SCORAD classification, and no participant with moderate/severe AD worsened in the final assessment, which did not occur in the CG. This data suggests a possible positive effect of the messages sent. Approximately 40 % of patients in the GC, both with mild and moderate/severe AD, experienced worsening in their severity, probably due to the relapsing nature and multifactorial pathophysiology of AD. Both groups showed a significant reduction in the EASI, with no difference in the comparison between the groups.

The improvement in the severity of moderate/severe AD was more significant in the EG only according to the SCORAD. In this study, this may be associated with the subjectivity of this tool, which asks patients to rate their nocturnal and daytime pruritus in the last 48 h at the time of the assessment, which is not the case with the EASI.

Singer et al. conducted a randomized controlled clinical trial with 30 patients to evaluate the effect of sending daily educational text messages about atopic skin care on reducing the EASI score. They observed no significant difference in the severity of AD between the groups, but the control group received only outpatient advice and no messages.^11^ Pena et al. performed a pilot study with 25 adults and adolescents over 14 years of age with AD, who received daily messages about skin care for six weeks. They aimed to evaluate the effect of the intervention on adherence to treatment, self-care behaviors, the severity of AD, and the QoL of participants. All the parameters evaluated showed statistically significant improvements, but the authors did not use a control group and all participants received messages.^23^ In the present study, the CG received messages about health in general, which allowed us to better compare the two groups and understand whether the content of the messages influenced the result.

The authors observed a statistically significant improvement in the QoL of children and guardians only in moderate/severe AD cases in the EG (*p* = 0.01). Educational interventions may have a greater effect on the QoL of children with a more severe condition when caregivers feel a greater need to intensify care for better control of the disease and are more attentive to the guidance provided.

The literature is scarce in analyses similar to this study, which hinders the comparison of the results found. Studies with heterogeneous methodologies and educational interventions for children and guardians aimed to evaluate their effect on QoL, but none used text messaging. Schuttelaar et al. in the Netherlands with group and individual sessions conducted by the nursing team,^24^ Weber et al. in Brazil with biweekly group sessions for six months,^18^ Pustisek et al. in Croatia with single lectures and written materials,^5^ and Liang et al. in China with videos and printed materials for home^22^ also observed improved QoL in the intervention group.

The improvement in the severity of AD in children in both groups during this study may suggest the positive effects of educational interventions in general, not only those specific to AD. Messages about general health remind guardians of the importance of the skin care recommended in the consultation and positively affect adherence to treatment. Due to the multifactorial etiology of AD, alerting them to the importance of a healthy lifestyle, with a balanced diet and physical exercise, also led to better control of the disease and was a way of reminding them to intensify care in general for their child.

Moreover, receiving a message from the medical team, regardless of its content, may have induced the family to implement the care recommended in the consultation, acting as a reminder of the need to modify hygiene measures and avoid triggering factors. The messages, regardless of their content, strengthened the doctor-patient relationship, showing the attention of the health team and their concern for children's health, allowing better adherence to treatment with a consequent improvement in QoL. The literature does not provide a known scientific term to define this effect, therefore the authors suggest the Teleremembering Effect, defined as the action or effect of remembering again, reminiscing, or recalling a previously imposed or prescribed conduct or action after receiving an electronic text message. Moreover, more frequent consultations may have influenced adherence to treatment.^25^

This study is limited by the small number of patients evaluated. Besides the severity of AD and the treatment used, other variables could affect the effectiveness of educational measures, such as the socio-economic level of the family. Moreover, a final assessment of the study participants’ perceptions of the content and frequency of the messages received, and their possible effects would have been ideal.

Text messaging was a feasible and economically viable measure that could be an adjunct to treatment, improving the QoL of children with AD, especially in moderate/severe cases, and their guardians. This action allowed the health team to provide quality information to the families, demystifying wrong behaviors and beliefs and, consequently, reducing severity. Finally, the authors highlight the need for further studies on the effectiveness of educational interventions in AD.

## Funding sources

No funding was received to assist with the preparation of this manuscript.

## Conflicts of interest

The authors declare no conflicts of interest.

## Appendix Supplementary materials

Image, application 1

## Footnotes

Supplementary material associated with this article can be found in the online version at doi:10.1016/j.jped.2024.07.003.
