# Supplementary material for: WhatsApp and atopic dermatitis: a clinical trial
Source: J Pediatr (Rio J). 2024 Aug 8;101(1):67–73. doi: 10.1016/j.jped.2024.07.003 (PMC11763882; doi:10.1016/j.jped.2024.07.003)
Supplement: Supplementary file 1 [file mmc1.docx]

**JPED-D-23-00486**

**SUPPLEMENTARY MATERIAL**

**Table 1** List of messages sent to the experimental and control groups.

- 1. Experimental group messages

| **1^ST^ WEEK**  Good morning! Over the next 4 weeks you will receive messages about childhood health. These messages will be sent automatically and should not be answered, okay? Thank you for your participation and we hope you enjoy the content! Let’s go to the first message!   1. The most important treatment for Atopic Dermatitis is the use of moisturizing creams in large quantities and several times a day. 2. Sleeping for a child with Atopic Dermatitis can be very bad because of the itching. Try telling stories and playing calming music to reduce restlessness before bed. 3. Did you know that Asthma, Rhinitis, and Atopic Dermatitis are diseases in the same family and often go hand in hand? 4. Remember to always moisturize! The more hydration... The fewer Dermatitis flare-ups! ” 5. Atopic Dermatitis is a disease in which the person is born without the skin’s natural moisture, so the skin is drier and has less protection. 6. Making moisturizing a pleasant experience for the child makes it easier to apply the cream. Try putting on some fun music and drawing pictures with the cream on your child’s body! 7. Have you noticed that synthetic and wool fabrics irritate the skin more and cause more itching? Prefer cotton clothes so as not to irritate your child’s skin. |
| --- |

| **2^ND^ WEEK**   1. Atopic Dermatitis works like a rollercoaster: there are ups and downs! Don’t lose hope in times of flare-ups! 2. Did the child’s skin appear red and peeling? Remember to use the ointment as instructed at your appointment! 3. Long baths dry out the skin and cause more itching. Keep bath time from 5 to 10 minutes. 4. Good morning! Let’s fight Atopic Dermatitis today? How about achieving the goal of moisturizing your child’s skin 3 times a day? Let’s go! 5. Did you know that the itchiest areas caused by Atopic Dermatitis are usually the creases in front of the elbow, behind the knee, and on the neck? But sometimes it can affect the whole body. 6. Don’t skimp on moisturizer! Each area of your child’s body should receive a generous amount of cream. 7. Hot baths remove moisture from the skin, so after a hot bath, the skin becomes drier and itchier. |
| --- |

| **3^RD^ WEEK**   1. Sweating can make itching worse. On very hot days, try spraying cold chamomile tea on your child’s skin. 2. How about doing something different today? Before applying moisturizer to your child’s skin, talk to them and explain the importance of applying the cream. 3. Did you know that even the labels on clothes, underwear, and panties, when they’re not made of cotton, can irritate your child’s skin? 4. Remember to always keep your child’s nails trimmed to prevent them from hurting the skin when they scratch. 5. A 200ml jar of moisturizer should last an average of one week. Is your jar lasting long? Maybe you’re using too little moisturizer, keep an eye on it! 6. Remember that warm, quick baths are ideal for controlling dermatitis! 7. How about applying the cream when your child is scratching? Besides moisturizing, it also helps relieve itching! |
| --- |

| **4^TH^ WEEK**   1. Have you ever tried talking to your child about how he or she feels at school because of Dermatitis? Explain that there’s nothing to be ashamed of and that moisturizing their skin is an important step in controlling the disease! 2. Remember: quick baths, and then the more you moisturize, the less it will itch! 3. Did you know that the emotional aspect of the child and family influences Atopic Dermatitis? When children are very stressed or anxious, they may feel itchier. Encourage your child to do activities they enjoy, this reduces nervousness! 4. Colored and perfumed shampoos and soaps cause more itching in children with Atopic Dermatitis! Only use the products your doctor has recommended. 5. Avoid using negative words (“ugly,” “horrible,” or “weird”) to refer to your child’s skin! Acceptance of any illness begins at home. 6. How is the plan to moisturize your child’s skin 3 times a day going? 7. As the years go by, Atopic Dermatitis becomes milder, that is, the flare-ups become less frequent and a smaller part of the body is affected. Have hope and focus on treatment! |
| --- |

- 1. Control group messages

| **1^ST^ WEEK**  Good morning! Over the next 4 weeks you will receive messages about childhood health. These messages will be sent automatically and should not be answered, okay? Thank you for your participation and we hope you enjoy the content! Let’s go to the first message!   1. Did you know that a child is very different from a “small adult”? Children need nutrients in their diet to grow and develop. That’s why a healthy diet is essential. 2. Did you know that contact with electronic devices before going to bed can disturb sleep during the night? Try to prevent your child from using these devices for at least 2 hours before going to bed. 3. Remember that boxed or packet juices don’t have good nutritional quality and are high in sugar. Whenever possible, choose natural juices! 4. As you may know, children up to 6 months old should be monitored every month by their pediatrician. But did you know that all children must be accompanied at least once a year until they turn 18? 5. Children under 6 months old should not sunbathe. And above that age, always with sunscreen, okay? 6. Did you know that giving shelf-stable milk (cow’s milk) to a child under 1 year old increases the chances of anemia, malnutrition, and allergies? 7. Encourage your child not to keep secrets from you! That way, when something upsets them, they’ll know who to look for! |
| --- |

| **2^ND^ WEEK**   1. Did you know that half of your child’s lunch or dinner should include salad and vegetables??? 2. Chocolates, cookies, chewing gum, and lollipops greatly increase the chance of tooth decay and obesity in your child. Always choose fruit over sweets! 3. Have you ever heard the saying “children who don’t sleep don’t grow up”? Oddly enough, it is true. A substance responsible for bone growth is released at night during the child’s sleep. 4. Did you know that exposing children to cell phones, tablets, or television before the age of 2 greatly increases the risk of them needing to wear glasses? 5. Always keep an eye on what your child is doing on the internet! It’s the best way to avoid contact with malicious people. 6. Did you know that letting someone else breastfeed your child carries serious risks for them? Many diseases can be transmitted through someone else’s milk. 7. Did you know that obese children often become obese adults? |
| --- |

| **3^RD^ WEEK**   1. Did you know that sunscreen shouldn’t only be used when your child goes to the park, beach, or swimming pool? The right thing to do is to use sunscreen every day, even on cloudy days! 2. Don’t give up on offering your child healthy food just because they didn’t want it the first time. It takes time for children to get used to a new taste. For example: does your child not like carrots? Offer grated carrots one day, boiled carrots the next, mashed carrots the next, and so on. 3. The more liquid children drink at dinner or lunchtime, the less they eat! Try offering drinks only after meals. 4. Negotiate with your child how much time they spend on the computer or video games. Make an effort to keep this commitment every day. How about a maximum of 1 hour a day? 5. Teach your child to love animals and preserve nature! 6. Did you know that boxed juice from the market has more sugar than a soft drink? Prefer natural juices. 7. Play relaxing music and tell your child bedtime stories. This helps them to sleep soundly! |
| --- |

| **4^TH^ WEEK**   1. Does your child not like vegetables? Check out these tips for creating fun food: <http://delas.ig.com.br/filhos/2012-04-26/aprenda-a-fazer-comida-divertida-para-as-criancas.html> 2. Did you know that children who have a routine have better health? Try creating some habits little by little, such as the right time to go to bed, take a shower, have lunch, eat dinner, and do chores. Don’t forget the fun times too!!! 3. At breakfast, lunch, and dinner time, how about turning off the TV and cell phone games to have a meal with your child and talk about how your day has been or will be? 4. Did you know that children can also have high blood pressure? Therefore, avoid seasoning food with too much salt. Replace it with garlic, onion, parsley, and other natural spices. 5. Up to 6 months old, the only food a baby needs is breast milk or age-appropriate formula. Juices, tea, and even water take the place of milk in the stomach. 6. Physical exercise is essential for the health of children and adults. Did you know that playing tag or hide-and-seek with your child is beneficial for both of you? 7. Give your child at least one type of fruit and vegetable every day! |
| --- |
